# A traditional Japanese-style salt field is a niche for haloarchaeal strains that can survive in 0.5% salt solution

**DOI:** 10.1186/1746-1448-3-2

**Published:** 2007-03-09

**Authors:** Tadamasa Fukushima, Ron Usami, Masahiro Kamekura

**Affiliations:** 1 Bio-Nano Electronics Research Centre, Toyo University, 2100 Kujirai, Kawagoe, Saitama 350-8585, Japan; 2 Department of Applied Chemistry, Faculty of Engineering, Toyo University, 2100 Kujirai, Kawagoe, Saitama 350-8585, Japan; 3 Halophiles Research Institute, 677-1 Shimizu, Noda, Chiba 278-0043, Japan

## Abstract

**Background:**

Most of the haloarchaeal strains have been isolated from hypersaline environments such as solar evaporation ponds, salt lakes, or salt deposits, and they, with some exceptions, lyse or lose viability in very low-salt concentrations. There are no salty environments suitable for the growth of haloarchaea in Japan. Although *Natrialba asiatica *and *Haloarcula japonica *were isolated many years ago, the question, "Are haloarchaea really thriving in natural environments of Japan?" has remained unanswered.

**Results:**

Ten strains were isolated from a traditional Japanese-style salt field at Nie, Noto Peninsula, Japan by plating out the soil samples directly on agar plates containing 30% (w/v) salts and 0.5% yeast extract. They were most closely related to strains of three genera, *Haladaptatus*, *Halococcus*, and *Halogeometricum*. Survival rates in 3% and 0.5% SW (Salt Water, solutions containing salts in approximately the same proportions as found in seawater) solutions at 37°C differed considerably depending on the strains. Two strains belonging to *Halogeometricum *as well as the type strain *Hgm. borinquense *died and lysed immediately after suspension. Five strains that belonged to *Halococcus *and a strain that may be a member of *Halogeometricum *survived for 1–2 days in 0.5% SW solution. Two strains most closely related to *Haladaptatus *possessed extraordinary strong tolerance to low salt conditions. About 20 to 34% of the cells remained viable in 0.5% SW after 9 days incubation.

**Conclusion:**

In this study we have demonstrated that haloarchaea are really thriving in the soil of Japanese-style salt field. The haloarchaeal cells, particularly the fragile strains are suggested to survive in the micropores of smaller size silt fraction, one of the components of soil. The inside of the silt particles is filled with concentrated salt solution and kept intact even upon suspension in rainwater. Possible origins of the haloarchaea isolated in this study are discussed.

## Background

Salt, sodium chloride, has played a predominant role in human history. Hypersaline lakes and salt deposits have been natural resources of salt. People in hot and arid areas have also developed procedures to extract the salt from seawater using solar salterns. Seawater is concentrated gradually during flowing through increasingly concentrated pools until to crystallizer ponds. Japan is surrounded by sea, but there are no solar evaporation ponds, no salt lakes, or salt deposits. Japanese people have satisfied the need for salt by extracting salt from sea water using special methods through historic period until 1958 [1]. Since then, industrial salt production has been performed by concentrating sodium and chloride ions from seawater using the ion-exchange membrane electro-dialysis method, followed by boiling down of sodium chloride with vacuum evaporation apparatus (see the homepage of The Salt Industry Center of Japan [2]). Japan is still one of the largest importers of solar salt, mostly used in soda industry.

Haloarchaea, halophilic aerobic archaea, inhabit the hypersaline environments distributed throughout the world [3,4]. It was long thought that haloarchaea were not living in natural environments in Japan. As of January 17, 2007, 79 species have been validly published in the family Halobacteriaceae. In general, haloarchaeal strains require high salt concentration for growth and cell integrity. They, with some exceptions, lyse or lose viability in low-salt concentrations or distilled water, and water sensitivity or lysis-resistence has been a key differentiation criterion between halococci and other haloarchaea [5]. The most well known haloarchaea *Halobacterium salinarum*, for example, requires at least 2.5 M NaCl for growth and cells lose their morphological integrity instantaneously at less than 1 M NaCl [6]. Another representative, *Halococcus morrhuae *does not lyse in distilled water [5].

The first report on the isolation of haloarchaea in Japan appeared in 1980. A non-pigmented extreme halophile, strain 172, was isolated by enrichment culture of a sand sample with salt attached, collected on seashore in Japan [7,8]. This halophile was later designated as *Natrialba asiatica *[9]. The cells lysed in distilled water as in the case of most other haloarchaea.

**Figure 1:**
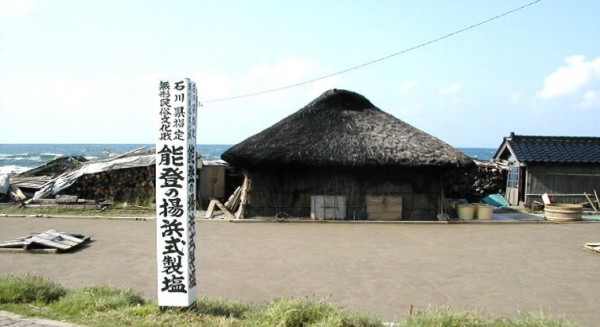
**Photograph of salt field at Nie**. The sign on left side reads (from up to down), Intangible Folk Cultural Property of Ishikawa Prefecture: Agehama-style Salt Production in Noto Peninsula. The sea behind the hut with thatched roof is the Sea of Japan. See the homepage of The Salt Industry Center of Japan [2] for details.

**Figure 2:**
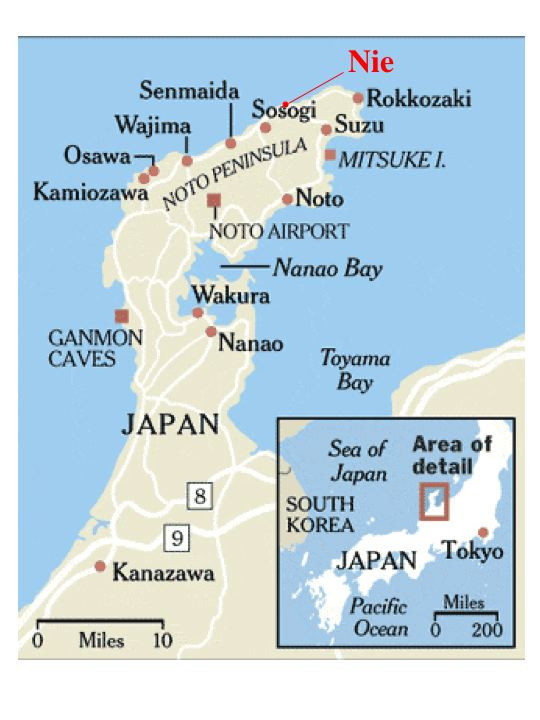
**A map of Noto Peninsula**. The Nie salt field resides 3 km east of Sosogi in the northern part.

In October 1985, scientists Horikoshi and Grant visited a salt field at Nie (Fig. 1) located on the coast of the Sea of Japan 3 km east of Sosogi of Noto Peninsula (see Fig. 2). They collected five soil samples from the salt field, and isolated seven strains on agar plates of a complex medium containing 4 M NaCl. Only one strain was shown to be extremely halophilic requiring at least 15% NaCl for growth [10]. It was reported that the cells of strain TR-1, designated as *Haloarcula japonica *[11], lysed upon suspension in 5% NaCl solution [12]. Although exact figures are not available for the Nie area, the statistics by Japan Meteorological Agency tell us that annual rainfall of Wajima City, only 22 kilometers west of Nie salt field, ranged from 1976 to 2560 mm during the last five years (2002–2006) with a mean of 2200 mm, compared to 1295 to1854 mm of Tokyo. It is known that the west coast of Australia, where solar salterns are operated, is arid with annual rainfall less than 300 mm (Bureau of Meteorology of Australian Government). In rainy season, the soil layer is sometimes flooded with heavy rainfall. Since 1986, however, no reports were published on the isolation of other haloarchaeal strains from natural environment in Japan. The question "Are haloarchaea really thriving in regions of Japan?" has not been answered yet.

The present study was designed in 2005, 19 years after the report by Horikoshi and Grant [12]. In this paper, the authors collected soil samples from the same salt field, and found that all soil samples taken from inside of the field contained haloarchaeal cells. We isolated 10 strains, analyzed their phylogenetic positions, and characterized their survival rates at low salt concentrations.

## Results

### Isolation of haloarchaea, growth patterns and Gram staining

**Figure 3:**
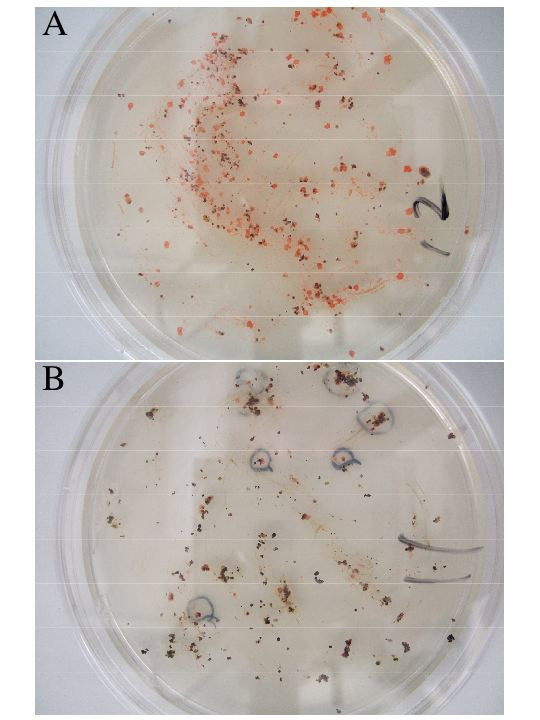
**Photographs of agar plates of samples No.12 (A) and No.11 (B)**. About 0.5 g of the sample was spread evenly on agar plate and incubated at 37°C for 30 days. Red colonies that appeared after 8 days incubation of plate No.11 (B) were marked with a black ink.

We plated out each sample directly onto 30% SW agar plates containing 30% salts and 0.5% yeast extract, spread the soil particles evenly on the surface of the plate with a spatula, and incubated at 37°C. After 8 days, many pink colonies appeared around soil particles on plates of sample No.7 and No.12 (Fig. 3A) and a few colonies on plate of No.11 (Fig. 3B). Further incubation up to 30 days resulted in formation of pink to red colonies on plates of all soil samples, at least a few per plate, that were taken inside of the field. Sample No.12 gave extraordinarily high numbers of colonies and most colonies seem not associated with black soil particles visible with naked eyes. A few white to beige colonies appeared on sand sample No.5 taken at seashore. No colonies appeared from three seawater samples. After repeated transfers on fresh medium, 10 strains were isolated and subjected to the following characterizations.

The 10 strains were cultivated in 25% SW liquid medium, and their growth patterns were observed at 37°C. Strains 10-1 and 14-1 grew very slowly, only after 2–3 days lag periods. On the other hand, strains 7-1, 7-2, 11-1, and 14-2 grew fast, with slight growth even after overnight culture. The final cell densities of the strain 7-1 and 7-2, however, were low, about one fifth of those of other 8 strains.

The 10 strains were pre-cultured in 25% SW liquid medium and streaked on agar plates of 25, 3, and 0.5% SW agar media, and incubated at 37°C for 10 days. No growth was observed on 3 and 0.5% SW agar plates.

Gram staining was done on stationary cells grown in 25% SW liquid medium. Strains 7-1, 7-2, 10-1, 10-2, and 11-2 stained negative, while strains 11-1, 13, 14-1, 14-2 were mixtures of cells that stained positive and negative. Most cells of the strain 12 stained gram positive.

### Phylogenetic analysis

**Figure 4:**
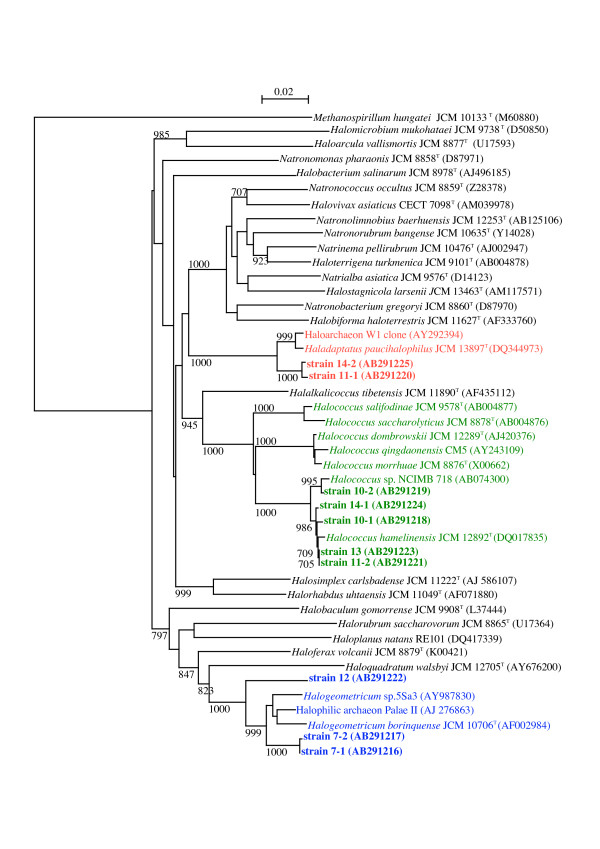
**A neighbor-joining phylogenetic tree inferred from 16S rRNA gene sequences**. Sequences of the 10 strains isolated in this study and those of the type strains of type species of all haloarchaeal genera validly published are comprised, as well as those of all species of the genus *Halococcus*, and sequences relevant to *Halogeometricum borinquense *and *Haladaptatus paucihalophilus*. The sequence M60880 of *Methanospirillum hungatei *was used as an outgroup. Bootstrap values greater than 70% in 1000 resamplings are displayed. Bar, 0.02 expected nucleotide substitutions per site.

The 16S rRNA gene sequences of strains 7-1, 7-2, and 12 showed highest similarities to that of *Halogeometricum borinquense*, 97.1, 97.0, and 94.6%, respectively. The sequences of strains 10-1, 10-2, 11-2, 13, and 14-1 were very close to that of *Halococcus hamelinensis *with 98.9–99.5% similarities. Strains 11-1 and 14-2 were most closely related to *Haladaptatus paucihalophilus*, with similarities of 97.5 and 97.6%, respectively. A phylogenetic tree reproduced by neighbor-joining method is shown in figure 4. We are in the process of fully characterizing these strains.

### Survival rates in low salt solutions

**Figure 5:**
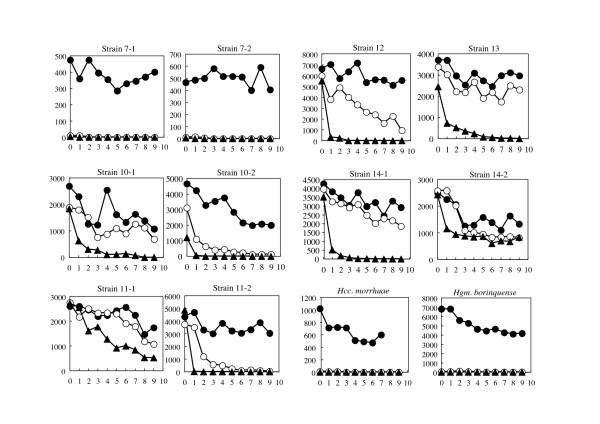
**Survival rates of the 10 strains in SW solutions**. The cells were suspended in the SW solutions containing 30% (●), 3% (○), and 0.5% (▲) salts, kept at 37°C, and viable cell numbers were counted every day. X-axis refers to days, and Y-axis refers to viable cell numbers. Note that the initial cell numbers vary depending on the strains.

In order to observe if the strains isolated from the salt field are tolerant to hypotonic solutions, three pellets of each strain cultivated in 25% SW liquid medium were suspended in sterile 30%, 3%, and 0.5% SW solutions, respectively, incubated at 37°C, and surviving cell numbers were measured every day. As shown in Fig. 5, at least 50% of the cells of all strains maintained viability in 30% SW solution for 9 days. Survival rates in 3% and 0.5% SW, however, varied considerably depending on the strains.

Two strains 7-1 and 7-2 died instantaneously upon suspension in 3% and 0.5% SW solutions, and the cells lysed leaving transparent solutions. Two reference strains, *Halogeometricum borinquense *JCM 10706^T ^and *Halococcus morrhuae *JCM 8876^T ^(= ATCC 17082^T^) also died instantly even in 3% SW solution. The cells of *Hgm. borinquense *lysed in 3 and 0.5% SW solutions, but the cell suspension of *Hcc. morrhuae *remained turbid (although red pigments had faded away), with even higher turbidity compared to the cell suspension in 30% SW. Microscopic observation of *Hcc. morrhuae *confirmed that the cells remained coccoid in 0.5% SW solution.

Viable cell numbers of strains 10-2 and 11-2 in 3% SW decreased to one tenth after 5 days, and cells died in 0.5% SW within a day. Strains 10-1, 12, 13, and 14-1 were slightly more resistant to 0.5% SW and maintained viabilities to some extents in 3% SW up to 9 days. The cell suspension of these strains in 3% and 0.5% SW remained turbid as in the case of *Hcc. morrhuae*, as was confirmed by microscopic observation.

Strains 11-1 and 14-2 possessed extraordinary strong tolerance to low salt conditions. About 20 and 34% of the cells, respectively, remained viable in 0.5% SW after 9 days incubation at 37°C. These cell suspensions again remained turbid, and no morphological change such as cell aggregation was observed.

## Discussion

### A question raised by the isolation of N. asiatica and H. japonica in Japan

*Natrialba asiatica *172, a non-pigmented extreme halophile, was isolated from sands on seashore [7]. At that time, and even nowadays, most scientists involved in research on extreme halophiles had tacit agreement that haloarchaea live only in highly saline environments and cells lyse instantaneously upon exposure to hypotonic solution in nature. In fact, scientists used to extract the purple membrane from *Halobacterium salinarum *or other haloarchaea by simply suspending cell pellets in distilled water or by dialyzing cell suspension against distilled water [13]. The cells of the strain 172 did lyse in distilled water. To our regret, the student who collected the sand sample did not record the sampling site, thus trials to isolate similar strains, and experiments on how many haloarchaeal cells were living there, and how the cells survived attack by rainfall were not possible. A red pigmented *Haloarcula japonica *TR-1 isolated from a soil sample of the salt field of Nie [10,14] was reported to lyse upon suspension in 5% NaCl solution [12]. A question that was raised naturally was "How did they survive in the soil of salt field that is attacked by heavy rainfall in summer and snowfall in winter?"

### Suggested presence of haloarchaea in low salinity environments

For many years, analyses of environmental DNA samples have suggested the presence of haloarchaea in non-saline environments. Jurgens et al. [15] amplified 16S rRNA genes from DNA extracted from Finnish forest soil, and detected sequences very closely related to that of *Halobacterium salinarum *(X96686, X96687). Diverse 16S rRNA gene sequences related to many genera of Halobacteriaceae were recovered from tidal marine and salt marsh sediments [16]. Rölleke et al. isolated DNA fragments encoding a gene of *Halobacterium *spp. from a biodegraded wall painting of 13th century in Austria [17]. More recently, the same group detected clones of 16S rRNA genes closely related to those of *Halococcus morrhuae*, *Natronobacterium innermongoliae *etc. [18]. It has been argued that weathering of stone and masonry results from physical, chemical, and biological processes, and practically all porous building materials subjected to rainwater and rising damp contain soluble salts, dispersed within the porous materials or locally concentrated. This results in the formation of deposits of crystallized salts, which may support growth of haloarchaea. The authors of the present paper believe that there may be a possibility that haloarchaeal cells have been carried to the deteriorated wall painting by either wind or feathers of waterfowls from salt lakes and salterns, for example those of southern Spain or Tunisia. The cells themselves may lyse and die, but their DNA molecules may remain within the porous materials for many years.

### Halobacterial strains recently isolated from low-salt environments

The design of this study was triggered by the following two interesting papers published in 2004. One paper was on isolation of haloarchaea from sediment samples of Colne salt marshes (Essex, UK) with pore-water of 3.2% salinity [19]. Another paper was on haloarchaea from microbial mats of the sulfide- and sulfur-rich Zodletone Spring (Oklahoma, USA) with a stream water salinity of 0.7 to 1.0% [20]. An isolate M6 was designated as *Haloferax sulfurifontis*, but strangely enough, the cells lysed within 24 hours in 1% NaCl [21]. These two papers stated that "a diverse community of haloarchaea exists in coastal salt marsh sediments" [19], and that "members of the *Halobacteriales *are not restricted to their typical high-salt habitats" [20]. Quite recently, a novel haloarchaea, *Haladaptatus paucihalophilus *was isolated from the Zodletone Spring, but from different part of the spring [22].

### Relatives of the Nie strains

In this study we have demonstrated that considerably high numbers of haloarchaea are thriving in the salt field, although further investigations on the diversity and seasonal fluctuation, etc. are needed. Phylogenetic analysis of the 10 strains showed that they were accommodated into 3 groups.

Strains 11-1 and 14-2, most closely related to *Haladaptatus paucihalophilus*, were extraordinary in their very strong tolerance of low salt conditions. Interestingly enough, pink-pigmented cells of *H. paucihalophilus *DX253^T ^isolated from soil samples of the Zodletone Spring mentioned above, remained viable in distilled water for up to 2 weeks, although quantitative survival rate was not shown [22]. Another strain W1 isolated from the Wareham salt marsh, Norfolk [19] is also closely related to *H. paucihalophilus*. This strain also did not lyse and remained viable in distilled water [23] (quantitative data not presented), and showed growth in an artificial seawater medium containing from 3.5% up to only 12% NaCl [23]. Strain 11-1, strain 14-2, and *H. paucihalophilus *gave good growth in media containing 23% or higher NaCl concentration, suggesting they constitute different species in the genus *Haladaptatus*.

Viabilities of strains 10-1, 10-2, 11-2, 13, and 14-1 in 3% SW decreased gradually, and cells suspended in 0.5% SW died more rapidly. The closest relative of these 5 strains *Halococcus hamelinensis *was isolated from stromatolites collected in Hamelin Pool, Shark Bay, Western Australia [24]. The salinity of the surface water in Hamelin Pool was twice that of normal seawater. It has been recognized that cells of *Halococcus *spp. are resistant to lysis in hypotonic solutions. The cells of type strain of *Halococcus morrhuae*, JCM 8876^T^, did not show morphological changes, but lost viabilities instantaneously upon suspension in 0.5 or 3% SW.

Strains 7-1 and 7-2 were most closely related to *Halogeometricum borinquense *ATCC 700274^T ^= JCM 10706^T ^isolated from a solar saltern of Puerto Rico. Although no description is given on the viability in hypotonic solution of the type strain, it requires at least 8% NaCl to grow [25]. Strains 5Sa3 and PalaeII were isolated from Maras saltern in Peruvian Andes [26] and the Crete Island, Greece, respectively, but their characterizations have not been published. Strain 12 that stained Gram positive, the only exception in the 10 strains, with the highest similarity to *Halogeometricum borinquense *(94.6%) may represent a novel taxon.

Although a few white to beige colonies appeared on sand sample No.5 taken at seashore, we believe they were those of halophilic strains of the family Bacillaceae as were isolated by Echigo et al. [27] from ordinary field soil samples and seashore sand samples.

### How do they survive in the salt field soil when attacked by heavy rainfall?

It is easy to assume that strains 11-1 and 14-2 are able to survive attacks by rainfall even if they are living as free cells, but how do strains 7-1 and 7-2 survive? Soil is made up of three components: solid particles, air, and water. The particles are classified by size as clay (less than 2 μm), silt (4 to 63 μm), sand (0.063 to 2 μm), gravel (2 to 64 mm), etc. Studies on field soil have demonstrated that cell numbers and microbial biomass were most concentrated in the smaller size silt and clay fractions, and microbial biomass is also mainly present in micropores (5 to 30 μm) [28]. A schematic illustration of soil particle is given in [29]. The numerous numbers of pink to red colonies observed on agar plate of sample No.12 (Fig. 3A) may be explained by assuming that the sample is composed of many numbers of silt particles hardly visible when covered with colonies originating from a few cells inside. It may be reasonable to speculate that the fragile strains 7-1 and 7-2 are surviving in the micropores of smaller size silt fractions, the inside of which is filled with concentrated salt solution and kept intact even upon suspension in rainwater. It is well known that methanogenic archaeal strains are strict anaerobes, and they die immediately when the colonies on agar slopes are exposed to air. The methanogens, however, survive in a paddy field soil samples broken mechanically and passed through stainless steel sieves of 100 μm mesh size. Once the soil particles, dried in air for several months were put into anoxic distilled water, initiation of methane production was observed [30]. This fact suggests that soil particles as small as 100 μm serve as good habitat for the fastidious microbes, including haloarchaea.

### Where did the Nie haloarchaea come from?

A frequent question concerns the source of the haloarchaea in solar salterns, man-made hypersaline environments. A Spanish group has reported that coccoid extreme halophiles were isolated from Mediterranean seawater samples collected 5 km offshore from Alicante where salterns have been operated [31]. A representative isolate survived in seawater for at least a month at room temperature. The authors [31] pointed out a possibility that the haloarchaea originated from the salterns. Another paper, however reported that *Halococcus *representatives were present in seawater samples in area where salterns or other hypersaline habits were not available [32]. In this context, it should be pointed out that many multi-pond solar salterns are operated commercially in the west and south-west coast of Korea confronting Yellow Sea, from which many halophilic bacteria have been isolated (for example, see [33]). Although the distance from the west coast of Korea to Noto Peninsula is more than 1000 km, a surface oceanic current, Tsushima Current, is running along the west coast of Japan. The Current is the northeastward-flowing branch of the Kuroshio (Japan Current) entering the Sea of Japan through the Tsushima-Korea Strait. Recent studies by tracking the tracer particles placed on estuary of west coast of Korea demonstrated that a number of the particles passed through the Strait and spread through the Sea of Japan in months (Tetsuo Yanagi, Kyushu University, Japan [34]). It may be speculated that some haloarchaea similar to the strains 11-1, 14-2 isolated in this study migrate from the solar salterns on the west coast of Korea, their original habitat, to the coast of Nie. The salt field at Nie has been operated for 400 years, and a huge volume of seawater has been sprayed over the soil. A similar but more difficult question has long been raised, "Where did the haloarchaea of the Dead Sea or the Great Salt Lake, for instance, come from?" The Lisan Lake, the precursor of the Dead Sea, existed between 70,000 and 15,000 years ago [35,36], very young in a geological and microbial evolution scale.

Echigo et al. [27] reported that many endospore-forming halophilic bacteria that were able to grow in the presence of 20% NaCl were inhabiting non-saline environments such as ordinary garden soils, yards, fields, and roadways in the area surrounding Tokyo. They discussed that those halophilic bacteria have been kept transported by Asian dust storm to Japan for thousands of years from the indigenous highly saline environments, such as salt lakes and the surrounding saline soils in Inner Mongolia, China. Why didn't they detect any haloarchaea or other halophilic bacteria of other families? Echigo speculated that cells of these halophiles would die sooner or later after they arrived at non-saline environments because of the hypotonic conditions caused by rainfall [27]. The authors of the present paper believe that there is a possibility that haloarchaeal cells trapped inside of silt particles, rather than as naked cells, are transported by the Asian dust storm and arrive at the Nie salt field, although in fairly low frequencies, and have survived and grown somehow in the soil particles. Attempts to trap haloarchaea from Asian dust are needed, as well as model experiments to investigate the fate of the individual fragile strains isolated in this study.

## Methods

### Outline of the salt field

The salt field at Nie of Noto Peninsula, Ishikawa Prefecture (Fig. 1 &2) was built in 1596. This is the only remnant of traditional salt fields that prospered throughout Japan until 60 years ago, and was registered as an Intangible Folk Cultural Property of Ishikawa Prefecture in 1992. A terrace of 13 m × 25 m was made by flattening the ground and hardening the base with clay of 15–20 cm thick. The terrace was located about 14 meters away from the beach of the Sea of Japan and about 3.5 meters above the sea level. In the morning of a hot and windy day in summer (from May to September), the terrace is covered manually with a layer of soil, with a thickness of 1.5 to 2 cm. Average diameter of soil particles is roughly 0.3 mm. About 450 liters of seawater carried in buckets from the Sea of Japan is sprayed over the layer of soil. By leaving for several hours with occasional mixing of the soil with a rake, water evaporates by sunlight and high wind, leaving soil granules incrusted with salts. The soil is then collected with a special rake to a wooden extractor well, into which seawater is poured to dissolve the incrusting salts. Seawater contains about 3.5% (by weight) dissolved minerals: 2.7% sodium chloride and 0.8% calcium, magnesium and sulfate ions (Salt Institute [37]). A concentrated salt solution (more than four times concentrated than seawater) drained from the bottom of the extractor well is put into a shallow pan and boiled by heating to obtain crystals of sodium chloride (see the homepage of The Salt Industry Center of Japan [2]). The soil in the concentrator is recovered and used repeatedly.

The salt concentration of aqueous part of the salt field, thus, fluctuates within a day from 3.5% of seawater to saturation just before water is evaporated completely. The concentration decreases to almost zero when attacked by heavy rainfall. A video of the actual field labor in the field is available [38] (the talks are in Japanese).

### Collection of samples and isolation of haloarchaeal strains

We visited the salt field on November 17^th^, 2005. At this season, the field was out of work, and most of the soil was already gathered to the center of the field and covered with a plastic sheet to prevent run-out by rain and snow fall during winter time. First of all, as controls, three samples (No. 1–3) of seawater left on hollows of rocks a few meters away from beach, and three sand samples (No. 4–6) of seashore were taken directly into sterile screw-capped 2 ml tubes. Eight soil samples (No. 7–14) were collected from different spots inside of the salt field. All samples were kept at 5°C until used.

The agar plates for the isolation of haloarchaea were prepared as follows; 2.0 g yeast extract (Difco) was dissolved in 4 ml of water in 500 ml medium bottle, 396 ml of 30% SW (Salt Water, prepared separately) and 8 g of Bacto agar (Difco) added, boiled for 15 to 20 min to melt the agar as completely as possible, and autoclaved. The composition of the 30% SW was as follows (per liter); 0.8 g NaBr, 0.2 g NaHCO_3_, 6.0 g KCl, 59.4 g MgSO_4_·7H_2_O, 41.5 g MgCl_2_·6H_2_O, 234 g NaCl, and 1.45 g CaCl_2_·2H_2_O, pH 6.5 (not adjusted). The CaCl_2_·2H_2_O was dissolved in small volume of water and added to the solution of all other ingredients with continuous stirring, and filled up to 1 liter. The proportions of salts are approximately the same as found in seawater, but ten times more concentrated.

About 0.5 g of the soil or sand samples was taken with a sterile micro-spatula and spread evenly on the agar plates with the spatula, while three drops of sea water samples were placed on agar plate and spread with a sterile spreader. The plates were incubated at 37°C in a plastic container to prevent desiccation. After incubation for 30 days, colonies separated enough from adjacent ones were picked up randomly and purified by serial dilution and plating out on fresh agar plates. Finally 10 strains, 7-1, 7-2, 10-1, 10-2, 11-1, 11-2, 12, 13, 14-1, and 14-2 were subjected to further characterization. The strains are now kept on agar slopes at 5°C and at -20°C in L-dried ampoules.

### Measurement of survival cell numbers in hypotonic solutions

The 10 strains and 2 reference strains, *Halogeometricum borinquense *JCM 10706^T ^and *Halococcus morrhuae *JCM 8876^T^, were inoculated into 150 ml Erlenmeyer flasks containing 20 ml of 25% SW medium, and shaken at 37°C for 9 days. Three aliquots of 1.5 ml of each culture were centrifuged at 15,000 × g and supernatants were discarded. Each cell pellet was suspended gently in 1.0 ml of 30%, 3% and 0.5% sterile SW solution (for example, 3% SW, approximately the same concentration as seawater, was prepared by diluting the 30% SW tenfold with distilled water), respectively, with a micro tip and incubated at 37°C for 9 days. Every day, 10 μl of each suspension was taken and diluted 10^-4 ^with SW solution of the same concentration, and 20 μl aliquot was spread on agar plates of 25% SW medium and incubated at 37°C. The agar plates used every day were prepared a day before. The numbers of colonies were counted after 1 to 3 weeks depending on the strains (see Results).

### PCR amplification of 16S rRNA encoding genes and sequencing

16S rRNA encoding genes of the 10 isolates were amplified by PCR with the following forward and reverse primers: 5'-ATTCCGGTTGATCCTGCCGG (positions 6–25 in *E. coli *numbering) and 5'-AGGAGGTGATCCAGCCGCAG (positions 1540-1521). DNA polymerase was Platinum Taq High Fidelity (Invitrogen). The PCR was done by 25 cycles, each of which consisted of denaturation for 20 s at 96°C, annealing for 15 s at 55°C, and polymerization for 2 min at 72°C. The amplified genes were cloned into pCR2.1 T-vector (Invitrogen) and sequenced using the Big Dye Sequencing Kit Ver. 3.1 (Applied Biosystems) by the ABI 310 DNA sequencer (Applied Biosystems). Sequencing primers used were 5'-ATTCCGGTTGATCCTGCCGG (positions 6–25 in *E. coli *numbering), 5'AGGAGGTGATCCAGCCGCAG (positions 1540-1521), 5'ATTGGGCCTAAAGCGTCCGTA (positions 563–585), -20: 5'-GGAAACAGCTATGACCATG (vector side's primer) and Rev: 5'-GTAAAACGACGGCCAGT (vector side's primer).

### Phylogenetic analysis

The 16S rRNA gene sequences of type strains of type species of all haloarchaeal genera that have been validly published (as of January 17, 2007) were obtained from DNA Data Bank of Japan (DDBJ). The sequences were aligned by using CLUSTAL X Multiple Sequence Alignment Program. The phylogenetic tree was reconstructed by neighbor-joining method [39] and was evaluated by 1000 bootstrap samplings.

## Authors' contributions

TF did characterization of the isolates, the gene sequencing and phylogenetic analysis. RU co-designed the work, and MK designed the work, did the field work, and wrote the manuscript.

All authors have read and approved the final manuscript.
